# Electrical Transport and Power Dissipation in Aerosol-Jet-Printed Graphene Interconnects

**DOI:** 10.1038/s41598-018-29195-y

**Published:** 2018-07-18

**Authors:** Twinkle Pandhi, Eric Kreit, Roberto Aga, Kiyo Fujimoto, Mohammad Taghi Sharbati, Samane Khademi, A. Nicole Chang, Feng Xiong, Jessica Koehne, Emily M. Heckman, David Estrada

**Affiliations:** 1 Micron School of Materials Science and Engineering, Boise State University, Boise, ID 83725 United States; 2 KBRwyle, 2601 Mission Point Blvd, Suite 300, Beavercreek, OH 45431 United States; 3 Department of Electrical and Computer Engineering, University of Pittsburgh, Pittsburgh, PA 15261 United States; 4 NASA Ames Research Center, Moffett Field, CA 94035 United States; 5 Air Force Research Laboratory, Sensors Directorate, 2241 Avionics Circle, Wright-Patterson AFB, OH 45433 United States

## Abstract

This paper reports the first known investigation of power dissipation and electrical breakdown in aerosol-jet-printed (AJP) graphene interconnects. The electrical performance of aerosol-jet printed (AJP) graphene was characterized using the Transmission Line Method (TLM). The electrical resistance decreased with increasing printing pass number (n); the lowest sheet resistance measured was 1.5 kΩ/sq. for n = 50. The role of thermal resistance (R_TH_) in power dissipation was studied using a combination of electrical breakdown thermometry and infrared (IR) imaging. A simple lumped thermal model (${\boldsymbol{\Delta }}{\bf{T}}={\bf{P}}{\boldsymbol{\times }}{{\bf{R}}}_{{\bf{TH}}}$) and COMSOL Multiphysics was used to extract the total R_TH_, including interfaces. The R_TH_ of AJP graphene on Kapton is ~27 times greater than that of AJP graphene on Al_2_O_3_ with a corresponding breakdown current density 10 times less on Kapton versus Al_2_O_3_.

## Introduction

Wearable technology is an emerging multi-billion-dollar industry that is made possible, in part, by advances in flexible and wearable electronic devices^1–4^. Conventional fabrication processes such as vacuum deposition, photolithography, and epitaxial growth of electronic materials tend to be complex, expensive, and incompatible with rapid prototyping^5–7^. Additive manufacturing techniques, such as inkjet printing, aerosol jet printing (AJP), and extrusion printing, are being explored as alternative fabrication methods for such sensor systems^8–12^. Direct write techniques offer a low-cost fabrication alternative due to the reduced material consumption while allowing for rapid customization and prototyping^9,12–14^.

Despite the rising popularity of printing techniques, there is a growing need for ink formulations and materials to meet the demand of the electronics industry. Printable, conductive metals like Ag and Cu have been widely studied, but their applications are restricted by their high cost and the rapid oxidation of Cu^9^. While conductive polymer inks provide low cost printing, their performance is limited by their low conductivities, and poor thermal and chemical stabilities. Carbon nanotubes (CNTs), have shown promise as an AJP compatible ink with significant mechanical flexibility and high mobility making them attractive for AJP applications^15,16^. Nevertheless, due to poor dispersion of CNTs in AJP compatible inks and the high cost of monodispersed solutions, the applications of CNTs remain limited for AJP printable devices^17,18^. One of the more promising nanomaterials for such applications is graphene, a two-dimensional (2D) hexagonal carbon structure with sp^2^ hybridized carbon atoms^19^. Due to its high specific surface area, high carrier mobility, and unique band structure, graphene has shown many promising properties and demonstrated breakthroughs in electronic related applications^20–23^. Graphene is also a promising sensor electrode material due to its flexibility and high electrochemical activity at defect sites^3,24–26^.

Inkjet printing of graphene has been well established^9,27,28^, and several groups have demonstrated inkjet printed graphene chemical^29^ and biological^30^ sensors. Graphene inks are typically produced through liquid phase exfoliation of graphite or chemical and/or thermal reduction of graphene oxide^31,32^. These processes typically result in submicron graphene crystal domains, and give rise to numerous point defects within the lattice, and closed-contour defects around the flake’s edge^33^. Under applied electrical bias, these defects result in highly localized electric fields which can be modified by absorbed molecules/target analytes. Combined with the high electrical conductivity and specific surface area of graphene, these defects enable highly sensitive graphene based sensors able to detect target molecules with parts per billion sensitivity in controlled environments^33^. Furthermore, as inkjet is typically a drop-on-demand process, the microstructure of inkjet printed graphene typically results in a well layered structure with varying amounts of porosity, depending on annealing conditions, ink properties, and the number of print passes. In this regard, graphene’s compatibility with AJP is less understood^8,9,34^.

While additive manufacturing is rapidly advancing the low-power sensor applications of graphene, the high-power and high-temperature applications of additively manufactured graphene based devices have received less attention. Such applications include temperature sensors, resistive heaters, thermal heat spreaders, high-current carrying interconnects, and ordnance fuze technology^35–39^. Substrate properties, microstructure, and thermal interfaces are likely to play a key role in limiting reliability and power dissipation in such applications. Previous studies have reported on power dissipation processes for mechanically exfoliated, chemical vapor deposition (CVD), and epitaxial grown graphene-based devices. However, power dissipation in printed graphene-based devices has yet to be explored^40–45^. This work, therefore, investigates the roles of microstructure and the substrate properties on power dissipation in AJP graphene interconnects. The information gained from this study is expected to provide new fundamental insights that will impact low-power and high-power applications of AJP graphene devices, as device models for both will require understanding the physical properties of such materials systems and printed devices.

## Results

### Graphene ink characterization

**Figure 1:**
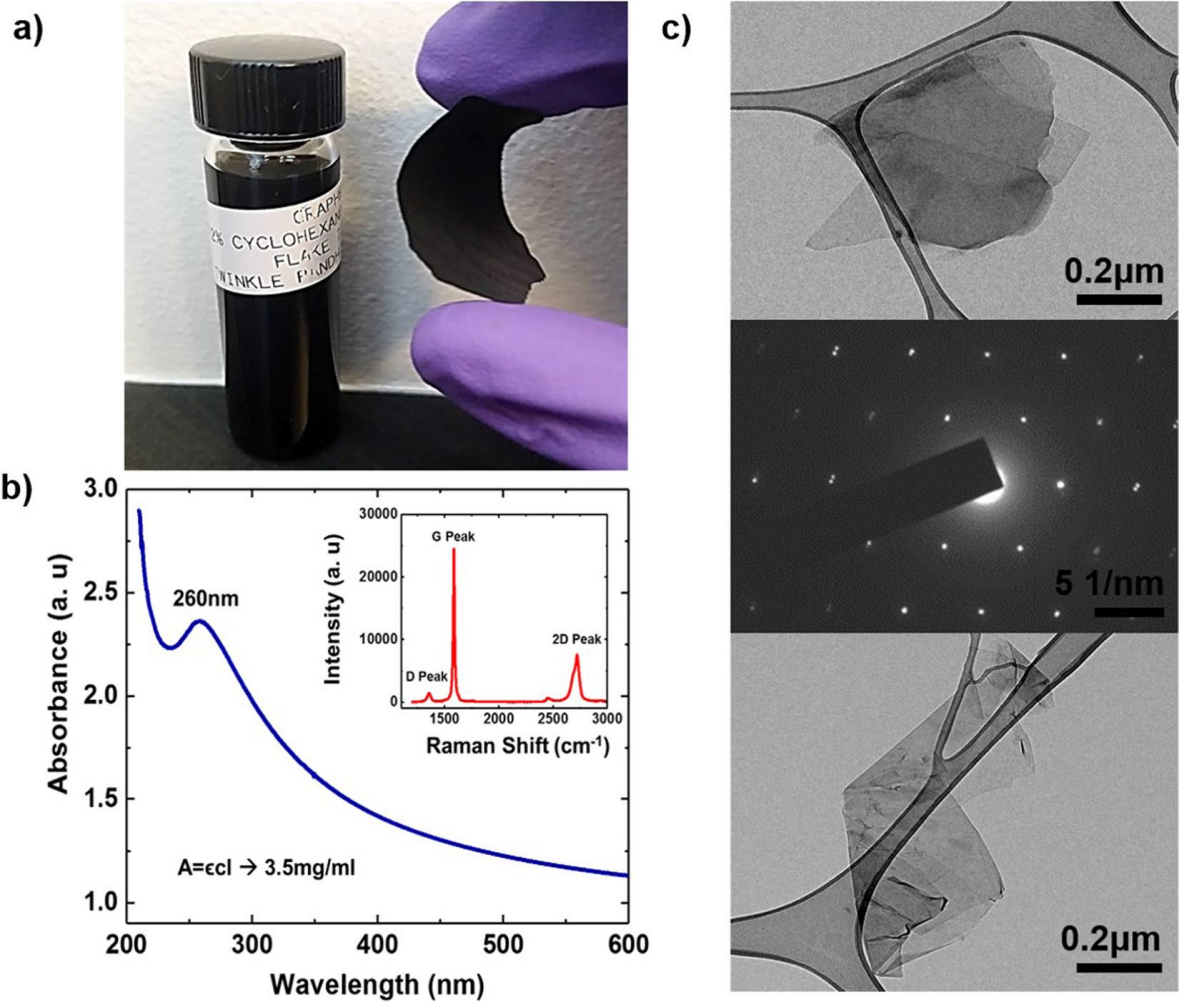
Graphene ink characterization (**a**) optical image of solvent exfoliated graphene/ethyl cellulose (EC) paper and AJP compatible graphene ink solution (**b**) UV-Visible absorption spectra is employed for quantifying the graphene flake concentration using the Lambert-Beer Law. Typical Raman spectra is seen (inset) for graphene/EC flakes on SiO_2_ (**c**) TEM images and diffraction pattern of graphene flakes used to compare observed lateral crystal dimensions to those calculated using Raman spectra and Cancado’s equation.

Graphene is obtained via solvent assisted exfoliation of bulk graphite, a process which has a relatively high yield of graphene flakes and is compatible with the ink synthesis processes. In order to develop highly-concentrated graphene ink, we use a combination of the processes reported in Jabari *et al*. and Secor *et al*.^8,28^. Bulk graphite powder was sonicated in ethanol and the stabilizing polymer ethyl cellulose to obtain suspended graphene flakes. The graphene flakes were then dispersed in a mixture of 92.5% cyclohexanone and 7.5% terpineol, which has been shown to be compatible with AJP (Fig. 1a)^8^. This resulted in an ink concentration of 3.5 mg/ml, which was quantified by UV-VIS absorption spectroscopy and Beer-Lamberts law (Fig. 1b). The ink viscosity of 3.6 cp was measured using a Cone Plate Wells Brookfield Viscometer. To image the individual graphene flakes, we dispersed the graphene in ethanol solution and drop casted onto TEM grids and a SiO_2_ coated Si wafer. These samples were then thermally annealed on a hotplate (250 °C for 10 min) and characterized with both TEM and Raman spectroscopy. Raman spectroscopy revealed the characteristic D, G and 2D peaks for graphene at 1350 cm^−1^, 1580 cm^−1^ and 2700 cm^−1^, respectively. The ratio of the D/G peak intensities (I_D_/I_G_) determines the quality (defect/disorder) of the graphene flakes. The I_D_/I_G_ peak ratio of 0.24 is lower than previously reported values (0.33–0.7), suggesting the exfoliated flakes are of higher quality with fewer defects^46^. TEM images show the graphene flakes vary in lateral size from ~50–200 nm. To correlate the TEM and Raman data, the I_D_/I_G_ peak ratio and 532 nm excitation wavelength was used in Cancado’s general equation^47^ to extract the crystal size (L_a_ ≈ 80 nm) of the graphene flakes. AFM characterization of the flakes shows the thickness (*t*_*g*_) ranges from monolayer to flakes with an average thickness of *t*_*g*_ = 16 nm +/− 15 nm (see Supplementary Information Fig. S1).

### Aerosol-Jet printed graphene interconnects

**Figure 2:**
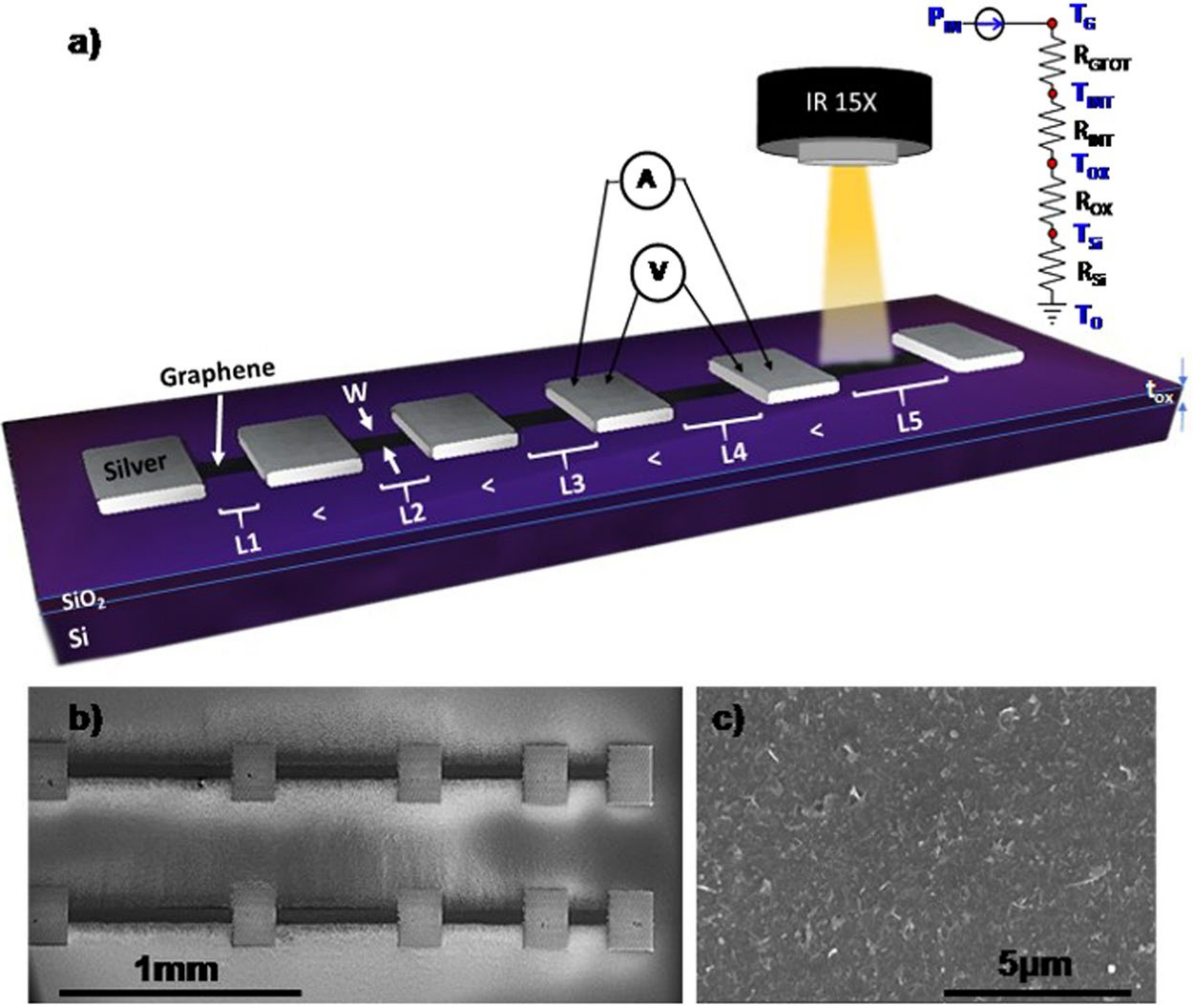
Investigating power dissipation of printed graphene interconnects with a combination of electrical breakdown and IR imaging. (**a**) Schematic of TLM experimental test structures of AJP graphene interconnects (increasing number of passes n = 5 to n = 50) with silver contact pads on Si/SiO_2_ (**b**,**c**) SEM images of the AJP printed/annealed graphene interconnects and a magnified SEM image to show the uniformity of the printed graphene.

The graphene interconnects and silver contact pads (Clariant Prelect TPS 35) were printed with an Optomec AJ-300 aerosol jet printer using the UA-max ultrasonic atomizer. The graphene print passes were varied from n = 5 to n = 50 and were deposited on SiO_2_/Si, Kapton and Al_2_O_3_ substrates. The graphene was printed in TLM test structures with 200 μm × 200 μm printed silver contacts (Fig. 2a)^48^. A recirculating bath temperature of 15 °C was used to stabilize the ink. After printing, the graphene lines were annealed for 60 min at 250 °C. The silver contacts were then printed on top of the graphene in a TLM structure. The SEM image of the AJP graphene TLM structure is shown in Fig. 2b. Figure 2c shows a magnified SEM image of the graphene line to observe the uniformity of AJP.

**Figure 3:**
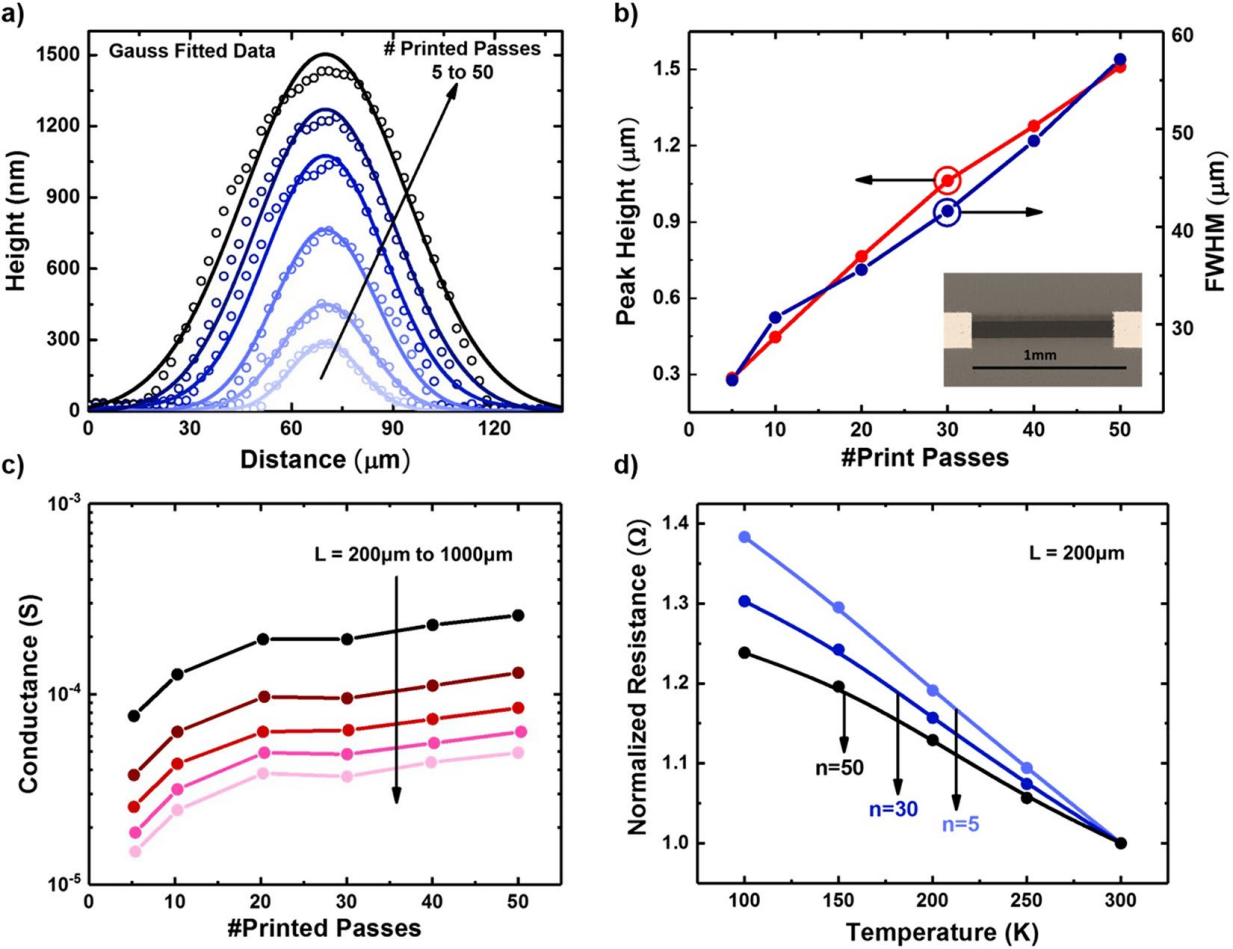
(**a**) Height profile of the graphene interconnect on Si/SiO_2_ is seen as a function of increasing number of print passes n = 5 to n = 50, shows a uniform deposition rate. (**b**) Full-width-half-max (FWHM) and peak height data extracted from the height profile provides additional support for the height correlation. (**c**) Electrical conductance of the graphene interconnect on Si/SiO_2_, for n = 50 pass line, with increasing length (L1 = 200 μm to L5 = 1000 μm). (**d**) Temperature-dependent measurements showing normalized resistance inversely proportional to temperature.

Using stylus profilometry, the change in height profile of the graphene interconnect was monitored as a function of increasing number of print passes. The height data seen in Fig. 3a shows a uniform deposition rate with an increase in height directly correlated to the number of passes. A similar height profile is observed for printed graphene interconnects on Kapton (see Supplementary Information Fig. S2a,b). The linear relation of the full-width-half-max (FWHM) and peak height data (Fig. 3b) extracted from the height profile provides additional support for this correlation. We note that while the FWHM of the graphene printed on Al_2_O_3_ substrates remains constant as the peak height increases with increasing pass number (see supplementary info Fig. S2d), suggesting the final morphology of the printed graphene interconnects can be influenced by the ink-substrate surface energy interactions^49^.

### Electrical scaling in AJP graphene interconnects

To measure the electrical properties of the printed interconnects, a 2-point probe (Keithley 4200 SCS) and TLM technique was used. As seen in Fig. 3c, for the 50-pass line on SiO_2_/Si, conductance decreases with increasing length (L1 to L5) as expected. Based on the TLM measurements the lowest sheet resistance was calculated as 1.5 kΩ/sq. for n = 50 at room temperature. Similar conductance profiles are seen for Kapton and Al_2_O_3_ (see Supplementary Information Fig. S3a,b). To understand the transport mechanism of the printed graphene, temperature-dependent measurements were performed. In Fig. 3d the normalized resistance is inversely proportional to the temperature. This observation agrees with the variable range hopping (VRH) model formerly established for graphene-based sensor devices; highlighting the potential to tune the graphene electrical transport properties from VRH to phonon limited conduction based on ink properties and printing parameters^21^. The electrical conductance increases by a factor of 30 based on the device dimensions and the number of print passes, which is in good agreement with literature^8^.

### Power Dissipation of AJP graphene interconnects

The overall power dissipation of a graphene device is dependent on the effective thermal conductivity and total thermal resistance of the system. Substrate material, interface thermal resistances, graphene quality, and device structure are a few of the factors that directly impact the total device thermal resistance^40,41,50^. To study this effect, a simple lumped model was developed that uses a combination of infrared (IR) thermal imaging and electrical breakdown thermometry supported by finite element modeling (FEM) using COMSOL multiphysics software^51–53^.

Simple lumped model: Similar to Ohm’s law (${\rm{\Delta }}{\rm{V}}={\rm{I}}\times {\rm{R}})$, the temperature rise (ΔT) in the graphene interconnects can be calculated as ${\rm{\Delta }}{\rm{T}}={\rm{P}}\times {{\rm{R}}}_{{\rm{TH}}}$, where P = I^2^ × R_EL_ is the dissipated power and R_TH_ is the total thermal resistance of the device. Here ΔT is comparable to ΔV, P is comparable to I, and R_TH_ = L/(κ_EFF_ × A) is the total thermal resistance and depends on the device dimensions and an effective thermal conductivity for the system (κ_EFF_). We note, that R_EL_ is the inverse of the device conductance (Fig. 3c), highlighting the potential to tune P based on print passes and device dimensions. To understand the limiting factors in power dissipation, R_TH_ is treated as a sum of the thermal resistances associated with the individual components of the system as illustrated in Fig. 2a. For the AJP graphene devices, R_TH_ is the sum of the graphene interconnect thermal resistance (R_GTOT_), the thermal interface resistance between graphene and the substrate (R_INT_ = 1/(g × A)), and the thermal resistance of the substrate (R_Sub_). For the SiO_2_/Si substrate R_Sub_ is the sum of the oxide thermal resistance (R_OX_ = t_OX_/(κ_OX_ × A)) and the silicon thermal resistance (R_Si_ = 1/(2 × κ_Si_ × A^1/2^). Here, t_OX_ = 90 nm, κ_OX_ = 1.4 Wm^−1^ K^−1^, κ_Si_ = 100 Wm^−1^ K^−1^, g is the graphene – SiO_2_ boundary thermal boundary conductance taken as 10^8^ Wm^−2^ K^−1^, and A is the area of the printed graphene interconnect (A = L × W)^54,55^. For samples printed on Kapton and Al_2_O_3_ the R_OX_ term is negligible and the substrate thermal resistances are simply (R_Sub_ = 1/(2 × κ_Sub_ × A^1/2^), where κ_Sub_ is the substrate thermal conductivity taken as 0.12 Wm^−1^ K^−1^ and 32 Wm^−1^ K^−1^ for Kapton and Al_2_O_3_ (sapphire), respectively^56,57^. Based on this model, a combination of IR microscopy and electrical breakdown thermometry can be used to quantify the heat spreading and estimate the “missing” R_GTOT_ associated with the printed graphene interconnects.

**Figure 4:**
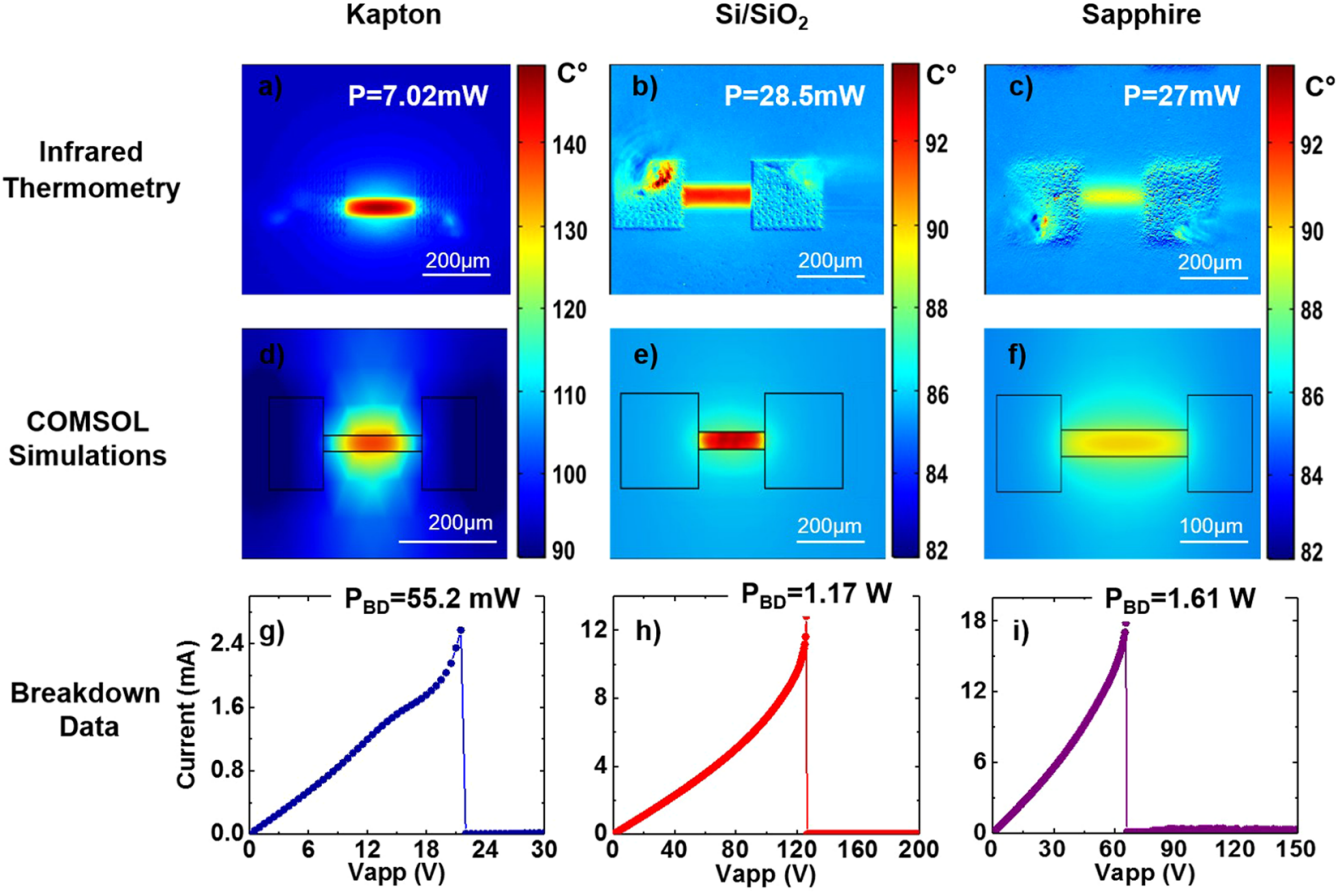
Power dissipation investigation of AJP graphene interconnects: Infrared (IR) thermal images of printed graphene interconnects with n = 20 print passes. (**a**) Kapton, (**b**) SiO_2_, and **(c)** Al_2_O_3_ (**d**–**f**) COMSOL simulation IR images to support the correlating experimental IR images seen above. The temperature scale bar is identical for both experimental and simulated results (**g**–**i**) Current vs. Voltage characteristics of AJP printed graphene interconnects on various substrates to extract power breakdown values.

IR Microscopy: The thermal profiles of the graphene devices were characterized under varying bias conditions. The background temperature T_o_ was set to 85 °C for a better signal to noise ratio over background IR emission. The thermal profile for graphene on Kapton (Fig. 4a) measured a temperature rise of 65 °C associated for an applied power of 7 mW. Comparatively, the temperature rise for the SiO_2_/Si is 10 °C for an applied power of 28 mW (Fig. 4b), and the temperature rise for Al_2_O_3_ is 5 °C for an applied power of 27 mW (Fig. 4c). These data illustrate the role of the substrate thermal properties on efficient heat spreading. For example, using the simple lumped model (ΔT = P × R_TH_), the high temperature rise at low power for Kapton results in a total thermal resistance of 9285 K/W compared to 350 K/W for SiO_2_/Si and 185 K/W for Al_2_O_3_.

Electrical Breakdown Thermometry and COMSOL: To quantify the R_GTOT_ contributions to R_TH_ a combination of electrical breakdown thermometry and COMSOL Multiphysics was used. Figure 4g–i show the corresponding I-V characteristics up to device failure for graphene interconnects printed on three different substrates. Failure of a Joule-heated device occurs when the temperature rise of the device from the background temperature (T_o_) of 85 °C exceeds the breakdown temperature (T_BD_), which is either the decomposition temperature of Kapton or the oxidation temperature of the graphene on SiO_2_/Si or Al_2_O_3_ (measured via TGA data, see Supplementary Information Fig. S4). The power values P of the graphene interconnects were measured up to device failure, which likely occurs when reaching the breakdown temperatures. Using the simple lumped model (Fig. 2a) and 550 °C as the oxidation temperature of carbon (verified by TGA data seen in Supplementary Information Fig. S4) for the graphene inks, the individual components of the thermal resistances for the graphene interconnects on SiO_2_/Si substrates can be quantified. Using this approach, R_GTOT_ is calculated as R_GTOT_ = R_TH_ − R_INT_ − R_OX_ − R_Si_. The total thermal resistance at the breakdown temperature is 397 K/W. This is only slightly higher than that calculated from low power and IR microscopy and is likely due to temperature dependences of the individual thermal resistances. At the breakdown temperature, R_INT_ = 1.1 × 10^−8^ m^2^ K/W, R_OX_ = 4.6 K/W, and R_Si_ = 42.3 K/W. Therefore, the total thermal resistance is dominated by R_GTOT_ = 349 K/W. A similar analysis for graphene interconnects on Al_2_O_3_ finds R_INT_ = 1.0 × 10^−8^ m^2^ K/W, R_Sub_ = 115.3 K/W and a slightly lower value of R_GTOT_ = 173 K/W. This lower R_GTOT_ value can be attributed to the physically thinner interconnect, which is approximately ½ as thick as N = 20 graphene interconnects printed on SiO_2_/Si substrates. Applying this model to the graphene interconnects on Kapton, with a melting temperature of 520 °C^56^ and a measured power of 55.2 mW, the total thermal resistance of the interconnect is found to be 7880 K/W. However, the calculated thermal resistance of the substrate is 3.67 × 10^4^ K/W, indicating significant heat transfer between the Kapton substrate and the supporting metal substrate during breakdown measurements.

**Figure 5:**
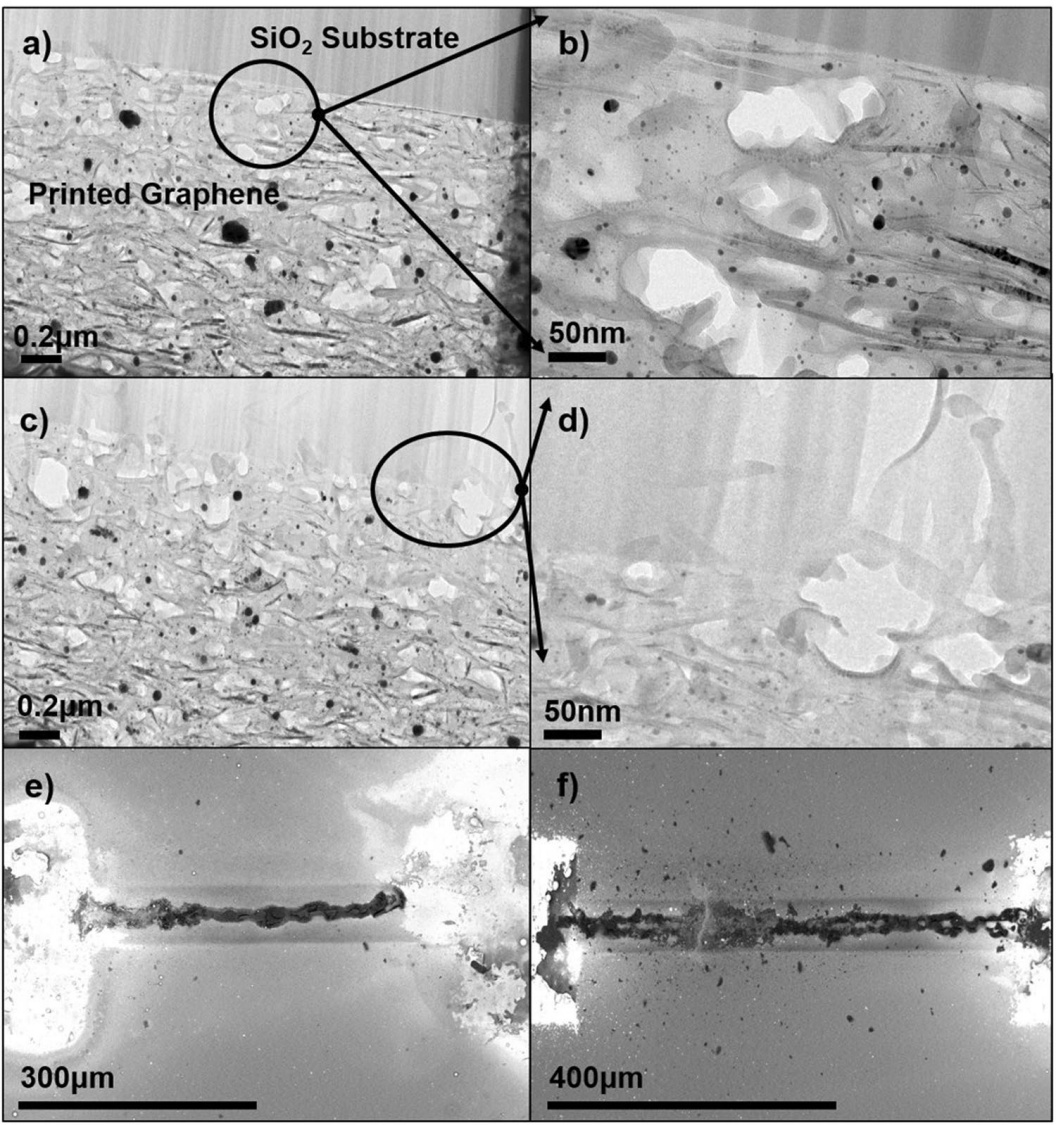
Investigating porosity and breakdown of the printed graphene interconnects: (**a**,**b**) and (**c**,**d**) Cross-section TEM images of the printed graphene interconnects on Si/SiO_2_ for n = 50 and (**e** and **f**) SEM images of different breakdown patterns of the printed graphene interconnects on Si/SiO_2_.

COMSOL multiphysics was used to further analyze the thermal spreading in these systems. Figure 4d–f show the corresponding COMSOL thermal images for the simulated device structure compared to the thermal images of the actual devices seen in Fig. 4a–c. The thermal profiles show that the experimental results for the imaged power dissipation are in good agreement with the computational results for all three substrates. Furthermore, the COMSOL simulation results can be used to analyze the temperature of each layer of the printed graphene device on SiO_2_/Si (the graphene interconnect layer, the interface layer, the oxide layer, and the silicon layer) in order to observe where the maximum power dissipation is taking place. The total thermal resistance of the interconnect on SiO_2_/Si was calculated to be 372 K/W, with the highest temperature value of 520 °C being reached within the graphene interconnect. From these calculations it can be concluded that the power dissipation is dominated by the graphene interconnect. The high thermal resistance of the graphene interconnects is likely due to several factors: the porosity of the printed interconnect, the high thermal resistance between graphene layers, and the general disorder of the constituent graphene nanoflakes that make up the interconnect^41,58^. Cross-sectional TEM imaging was used to quantify the porosity of the printed graphene interconnects on SiO_2_/Si and better understand the structure of AJP deposited graphene. Analysis of the TEM images seen in Fig. 5a–d indicate 15% porosity in the graphene interconnects. Furthermore, it can be seen that porosity at the graphene- substrate interface reduces the total area for heat flow across the interface, increasing the thermal interface resistance.

## Discussion

Graphene is known to have extremely good thermal conductivity (exceeding 2000 Wm^−1^ K^−1^)^59^ and high charge carrier mobility (~120,000 cm^2^ V^−1^ s^−1^)^60^, which makes it a desirable material for device applications. Nevertheless, the overall performance of graphene devices can be limited by power dissipation and the thermal resistance of the system^40^. Understanding the details of heat spreading (or Joule heating) in the system is important as it can limit the carrier mobility of graphene and the overall current density^41^. Effects of Joule heating are influenced by device structure, thermal transport across material interfaces, and the choice of substrate material^41^. While several studies have examined the impact of Joule heating in graphene devices fabricated using graphene obtained by various synthesis techniques such as mechanical exfoliation, epitaxial growth on SiC, and CVD growth on transition metal substrates, this is the first to do so for AJP printed graphene interconnects^40–42,59,61–64^. Our studies of power dissipation in AJP printed graphene interconnects indicate that power dissipation in AJP graphene is dominated by the graphene interconnect morphology for high thermal conductivity substrates, but can be limited by the substrate properties in the case of low thermal conductivity polymer substrates typically used for flexible and wearable electronics applications.

Before concluding we also wish to comment on the nature of the electrical breakdown of AJP deposited graphene interconnects. Electrical breakdown studies, which play a significant role in the elucidating the current-carrying capability of the interconnects, have also been investigated for graphene, CNTs, and CNF (carbon nanofiber)^51,53,58^. Due to different structure-property-processing correlations, vastly different breakdown patterns are expected under high electric fields. Generally, Joule heating and/or oxidation breakdown results in a physical break perpendicular to the direction of current flow, as seen in GNRs and SWCNT devices^65–67^. For CNTs, Joule heating may be the cause for breakdown at an early stage, but the main electric field and oxidation breakdown mechanism is driven by percolative pathways^51,68^. Moreover, Kitsuki *et al*. demonstrated the current induced breakdown of CNFs, and how the morphology of the graphitic layers comprising the CNFs play a significant role^58^. The cup-shaped features and voids observed can result in a quick break due to weak interlayer bonds of the graphitic layers.

This mechanism of CNFs breakdown can be applied to AJP graphene breakdown, due to their similar morphologies. This high porosity observed by the cross-section TEM images, gives rise to a high thermal resistance within the interconnect, and plays a significant role on the breakdown pattern of the graphene interconnects. Figure 5e,f show the breakdown patterns of n = 20 graphene interconnect on SiO_2_/Si used in this study. In both cases we find a breakdown pattern parallel to the direction of current flow. This type of breakdown pattern is likely due to the high porosity causing trapped gasses and solvents within the interconnect, as well as weak interlayer bonding of graphene flakes. As the device undergoes Joule heating, these trapped gases and fluids expand or vaporize, resulting in physical expansion and mechanical failure of the interconnect. This is particularly well captured in Fig. 5e, we do not see a break perpendicular to the direction of current flow. However, Fig. 5f shows both breakdown patterns suggesting a combination of a typical Joule heating and trapped gas/solvent driven breakdown for this device. Lastly, we note that the breakdown of AJP graphene on Kapton and Al_2_O_3_ was catastrophic with the substrate completely melting and destroying the graphene interconnect (for Kapton) and a complete oxidation and disintegration of the interconnect (Al_2_O_3_). (See Supplementary Information Fig. S5). However, further detailed analysis of the fundamental breakdown mechanisms is beyond the scope of this work and remains to be done in order to fully understand the physical nature of such breakdowns.

In summary, this study provides new insights into the electrical transport and power dissipation of aerosol-jet printed graphene interconnects. Graphene inks printed via AJP into TLM structures exhibited physical, electrical and thermal tunabililty based upon the number of print passes. Furthermore, electrical breakdown and infrared thermometry was performed to compare the power dissipation of the graphene printed interconnects on Kapton, SiO_2_/Si, and Al_2_O_3_ substrates. The combination of IR imaging and COMSOL simulation captured the Joule heating of the printed graphene and emphasized the role of device morphology and the substrate in power dissipation of printed graphene devices.

## Methods

### Preparation and characterization of Graphene ink

Similar to the processes described in Jabari *et al*. and Secor *et al*., graphene flakes were obtained by solvent assisted exfoliation of 50 mg/ml graphite powder in a suspension of 2% ethyl cellulose (EC) in ethanol using a Qsonica (Q125) probe tip sonicators for 90 min^8,28^. To remove the larger graphite flakes, the dispersion was centrifuged (Heraeus Megafuge 8 Centrifuge) at 4500 RPM for 30 min and the supernatant was collected immediately. In a 1:2 volume ratio, the collected supernatant and 0.04 g/ml aqueous solution of NaCl (Sigma-Aldrich, >99.5%) was centrifuged for 15 min at 4500 RPM, to facilitate the flocculation of graphene flakes. The resulting graphene/EC dispersion was dried overnight on a PTFE plate. To tailor the concentration and viscosity of ink to the AJP, the dried graphene/EC paper was then dispersed by sonication for 30 min, in a mixture of 92.5% cyclohexanone and 7.5% terpineol solution, followed by centrifugation at 4500 rpm for 15 min. The resulting ink concentration, as seen in Fig. 1a, is 3.5 mg/ml with a viscosity of 3.6 cP.

### AJP of graphene interconnects

The graphene interconnects were printed using an AJ-300 Aerosol Jet printer manufactured by Optomec. The atomizer utilized was the UA-Max ultrasonic atomizer. A recirculating bath temperature of 15 °C was used to help stabilize the ink temperature and prevent the output from being too solvent rich. The tool platen was heated to 65 °C to help ensure rapid drying of the ink once on the substrate. The printing nozzle was a 100 μm inner diameter ceramic and the mist tube material was polyethylene. The power applied to the atomizer was 48 W (48 volts at 1 amp). The sheath and atomizer flows were 50 and 20 sccm nitrogen respectively. The tool translation speed used was 1 mm/sec and the resulting single pass line width was measured to be ~50 μm. After printing any remaining solvent was driven out of the lines with a 100 °C bake for 10 min followed by a ramp to 200 °C bake under a nitrogen purge for 30 min to maximize conductivity of the printed features.

### AFM, TEM, and Raman Spectroscopy

For AFM, TEM and Raman characterization, the formulated graphene dispersion in ethanol/EC was diluted 1:10 volume ratio graphene/EC flakes in ethanol. The diluted dispersion was then drop-casted on to a TEM lacey carbon grid and tripled rinsed in isopropyl alcohol and dried with N_2_ Si/SiO_2_ wafers we used for AFM and Raman characterization. The TEM grid and SiO_2_/Si wafers were then annealed on a hotplate at 250 °C for 10 min, to remove the ethanol/EC residuals. AFM images were obtained using a Bruker Multimode 8 system. The images were collected with 1 µm × 1 µm scans, and particle characteristics were determined using Nanoscope Analysis software. TEM images were obtained using a JOEL JEM 2100 system, and the particles were characterized by ImageJ software. Lastly, the Raman peaks were obtained using Horiba LabRAM HR Evolution Raman microscope, with an excitation wavelength of λ = 532 nm. The spectrum was collected at a laser power of 25%, with magnification of 100x with 30 s exposure time, and between the range of 1000–3000 cm^−1^. Baseline subtraction was employed to clearly deduct the peak intensities. Cancado’s general equation^47^: ${L}_{a}(nm)=(2.4\times {10}^{-10})\,{\lambda }_{l}^{4}\,{(\frac{{I}_{D}}{{I}_{G}})}^{-1}$ was used to calculate the crystal size in the *a* axis (*L*_*a*_) of the graphene flake from the Raman spectrum.

### UV–VIS spectroscopy

Ultraviolet-Visible (UV-VIS) absorption spectroscopy (Cary 5000 G) was used to measure the optical absorbance of the graphene inks and quantify the graphene concentration. Using the Lambert-Beer law, A = αC_g_l, where A is the (absorbance), α (absorption coefficient), C_g_ (concentration of graphene), and l is (path length of the spectroscopy), a graphene concentration of 3.5 mg/ml was measured. The previously reported absorption coefficient at wavelength of 660 nm (α_660_ = 2460 L/g-m) was used in the calculations^8^.

### Thermogravimetric Analysis

In order to find the annealing temperature and oxidation temperature of the graphene, Thermogravimetric Analysis (TGA) was employed. A Netzsch TGA instrument was used to obtain the spectra of mass percent versus temperature. The mass of the dried graphene flakes (black) was analyzed as the temperature increased from 25 °C to 1000 °C at a heating rate of 5 °C/min in air. TGA analysis revealed the decomposition peak of surfactant, ethyl cellulose, is around 250 °C and oxidation/decomposition of the graphene is 550 °C (see Supplementary Information Fig. S4).

### Finite element model

In order to understand the heat dissipation and temperature distribution in our devices, we performed finite element simulations using COMSOL Multiphysics. In our thermal model, the bottom boundary of the substrate and the outmost surface of the silver pad (which were in contact with the probe) were kept at the ambient temperature under the isothermal boundary condition (*T* = *T*_ambient_). All other external boundaries were under the adiabatic boundary conditions, if they are thermally insulating. Thermal properties of the substrates are κ_OX_ = 1.4 Wm^−1^ K^−1^, κ_Si_ = 100 Wm^−1^ K^−1^, κ_Kapton_ = 0.12 Wm^−1^ K^−1^, and κ_Al2O3_ = 32 Wm^−1^ K^−1^. Thermal conductivities of graphene interconnect are assumed at κ_⊥_ = 2 Wm^−1^ K^−1^ and κ_||_ = 50 Wm^−1^ K^−1^ for cross-plane and in-plane directions, respectively. These values are significantly lower than those of pristine graphene because of the nature of printed graphene. The thermal interface resistance between graphene interconnect and substrates *R*_int_ were fitted to be between 1.0 × 10^−7^ m^2^ K/W to 1.0 × 10^−8^ m^2^ K/W.

### Data availability

The data generated and analyzed as part of this study are available from the corresponding authors upon reasonable request.

## Electronic supplementary material

Supplementary Information
