# Supplementary material for: Electrical Transport and Power Dissipation in Aerosol-Jet-Printed Graphene Interconnects
Source: Sci Rep. 2018 Jul 18;8:10842. doi: 10.1038/s41598-018-29195-y (PMC6052108; doi:10.1038/s41598-018-29195-y)
Supplement: Supplementary file 1 — Supplementary Information [file 41598_2018_29195_MOESM1_ESM.docx]

**Electronic Supplementary Material**

**Electrical Transport and Power Dissipation in Aerosol-Jet-Printed Graphene Interconnects**

Twinkle Pandhi^1^, Eric Kreit^2^, Roberto Aga^2^, Kiyo Fujimoto^1^, Mohammad Taghi Sharbati^3^, Samane Khademi^3^, A. Nicole Chang^1^, Feng Xiong^3^, Emily M. Heckman^4^, Jessica Koehne^5^, David Estrada^1^*

*^1^Micron School of Materials Science and Engineering, Boise State University, Boise, ID 83725, United States*

*^2^ KBRWyle, 2601 Mission Point Blvd, Suite 300, Beavercreek, OH 45431, United States*

*^3^Department of Electrical and Computer Engineering, University of Pittsburgh, Pittsburgh, PA 15261, United States*

*^4^Air Force Research Laboratory,* *Sensors Directorate*, *2241 Avionics Circle, Wright-Patterson AFB, OH 45433, United States*

*^5^ NASA Ames Research Center, Moffett Field, CA 94035, United States*

1. **Atomic Force Microscopy data of graphene flakes**


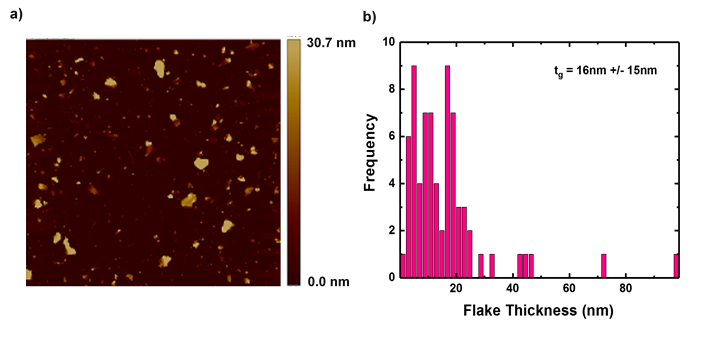


Figure S1. To characterize the graphene flakes, diluted graphene ink was drop-casted and annealed (at 100°C for 10 min) on Si/SiO_2_ wafer. (a) AFM scan of the dispersed graphene flakes was used to obtain particle statistics. Histogram of (b) flake thickness was extrapolated by using ImageJ.

1. **Height Profile for Kapton^TM^ and Al_2_O_3_ for printed graphene interconnects**


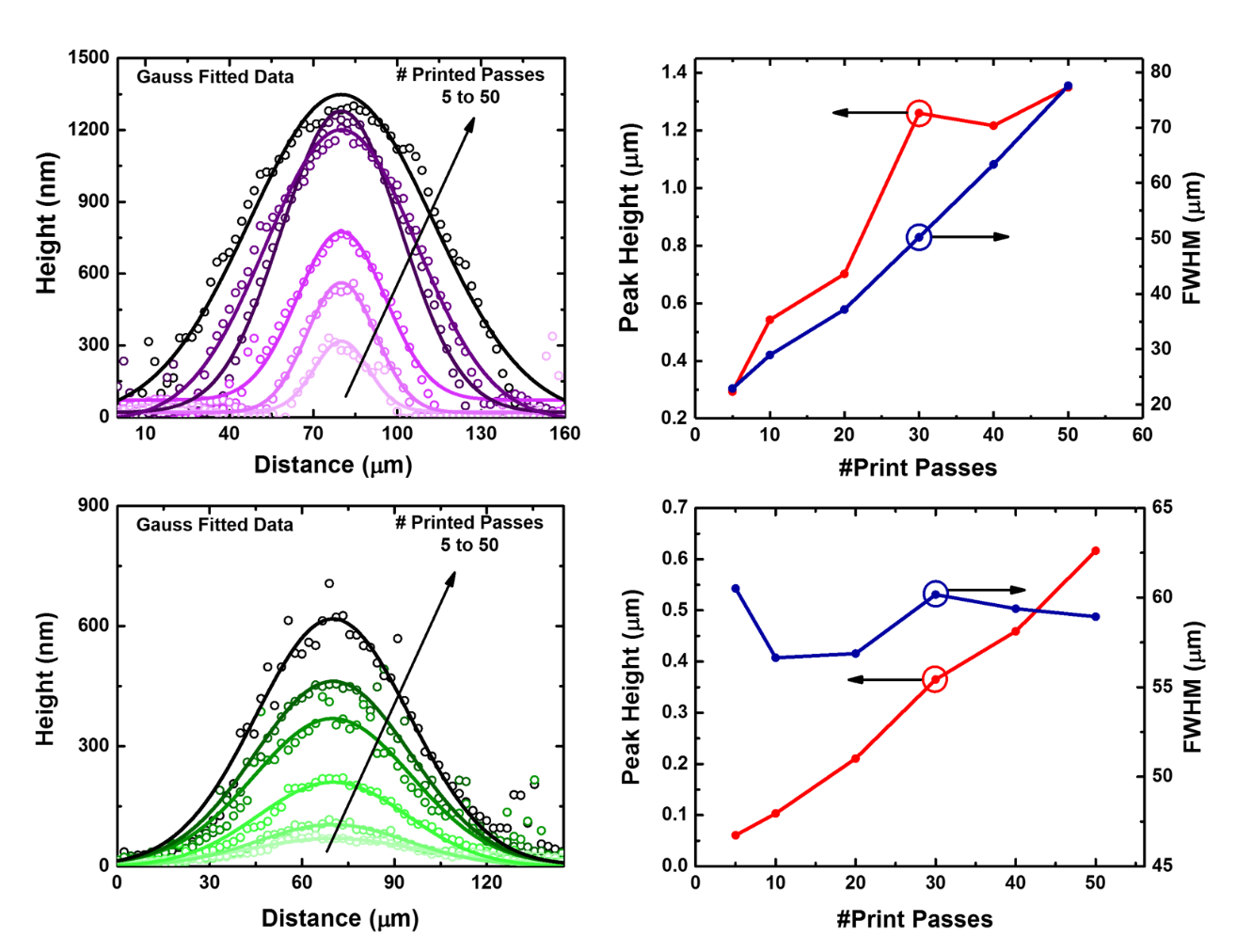


Figure S2. Stylus profilometry was used to extract height profile of the graphene interconnect was monitored as a function of increasing number of print passes on Kapton^TM^ (a, b) and on Al_2_O_3_ (c, d)

1. **Conductance data for printed graphene interconnects on Kapton^TM^ and Al_2_O_3_**


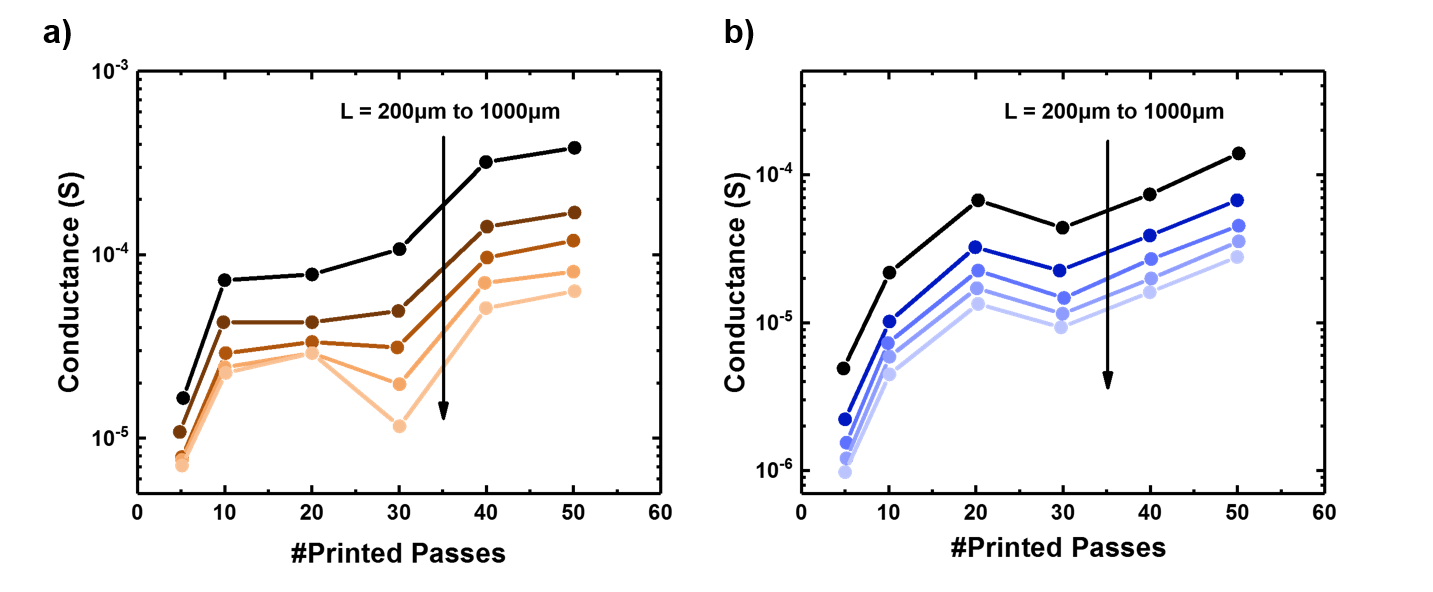


Figure S3. Conductance of the graphene interconnect was monitored as a function of increasing number of print passes on Kapton^TM^ (a) and on Al_2_O_3_ (b)

1. **Thermal Gravimetric Analysis for graphene flakes**


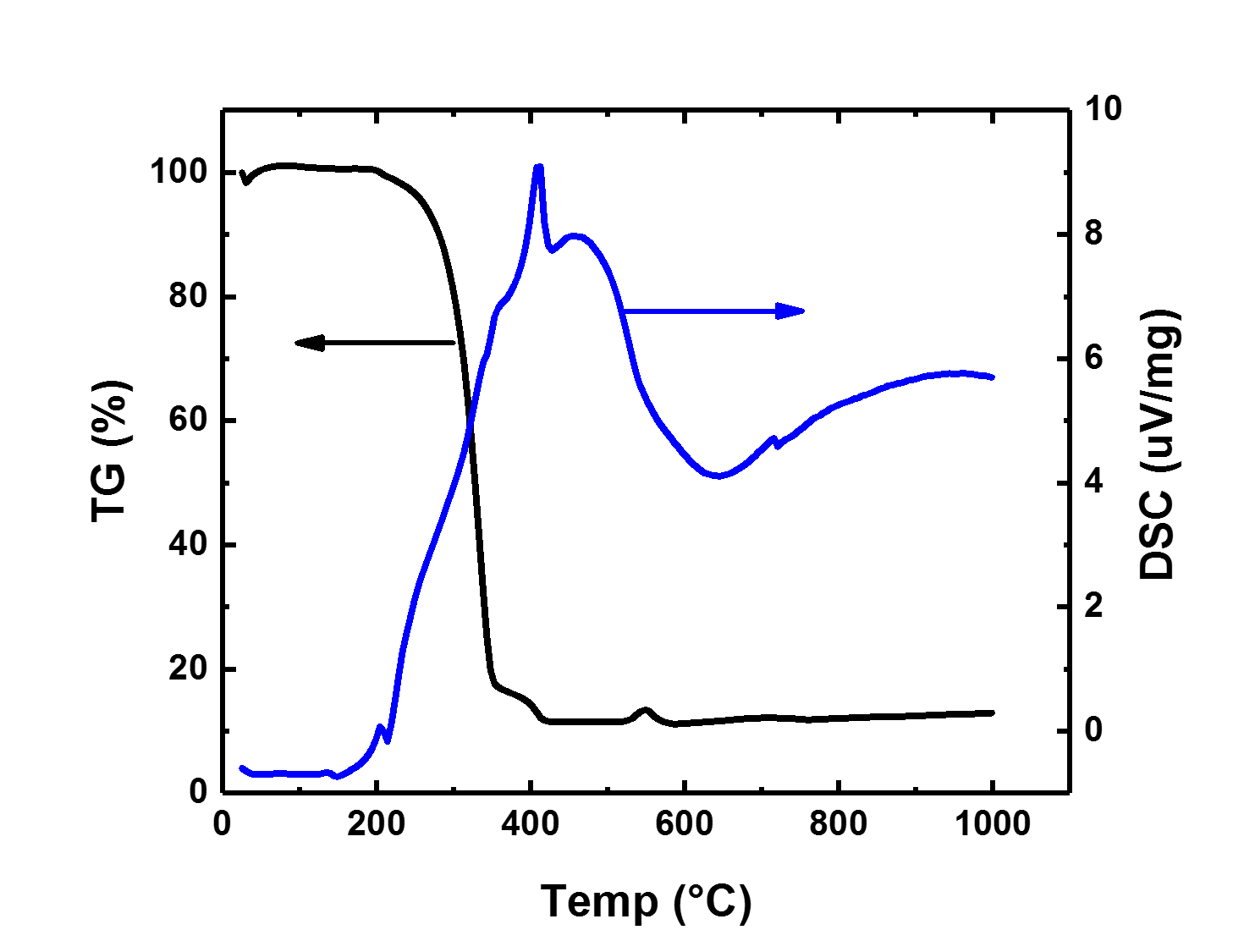


Figure S4. TGA (Netzsch instrument, at heating rate of 5°C/min in air) of the dried graphene flakes (black), showing mass as a function of temperature and (blue) the differential mass loss. The decomposition peak of surfactant, ethyl cellulose, is around 250°C and oxidation/decomposition of the graphene around 550°C

1. **Scanning Electron Microscopy images of the printed graphene on Kapton^TM^ and Al_2_O_3_**


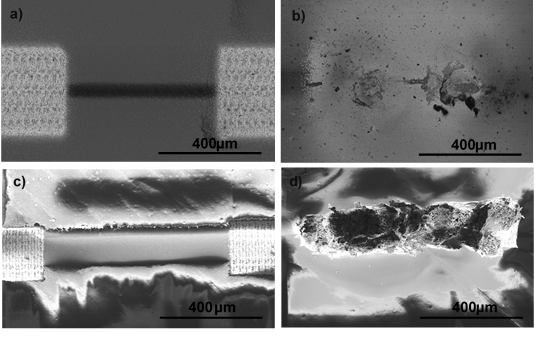


Figure S5. SEM images of the printed graphene interconnects on Al_2_O_3_ (a) and the corresponding breakdown image (b). Similarly, printed graphene interconnect SEM on Kapton^TM^ (c) and the corresponding breakdown image (d).

1. **Optical Images of Breakdown on Kapton^TM^**

**
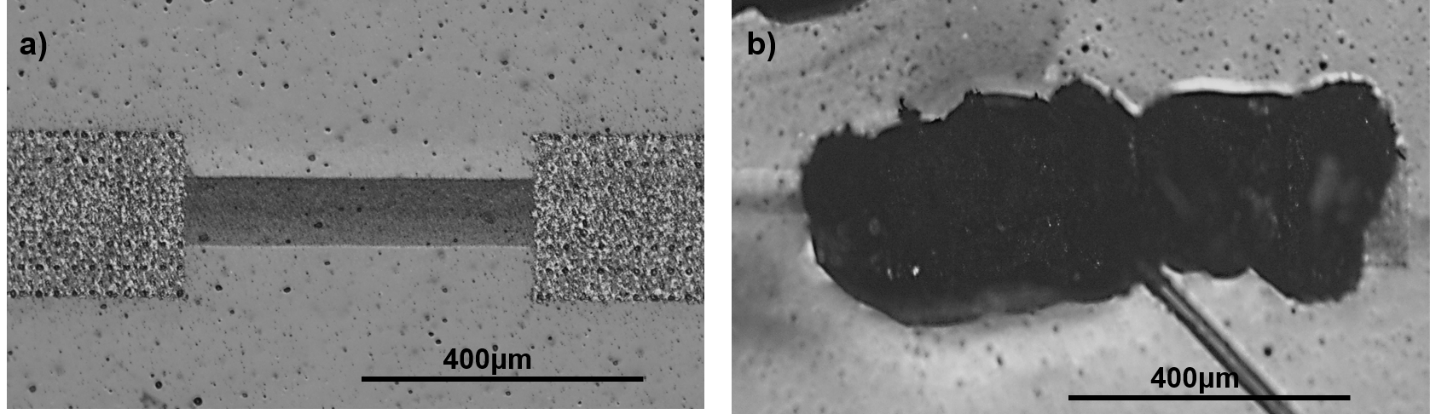
**

Figure S6: Optical images of an AJP graphene interconnect on Kapton^TM^ (a) and a similar device after breakdown (b).

1. **Additional power breakdown data for graphene interconnects on Kapton^TM^ , Al_2_O_3_ and Si/SiO_2_**

**
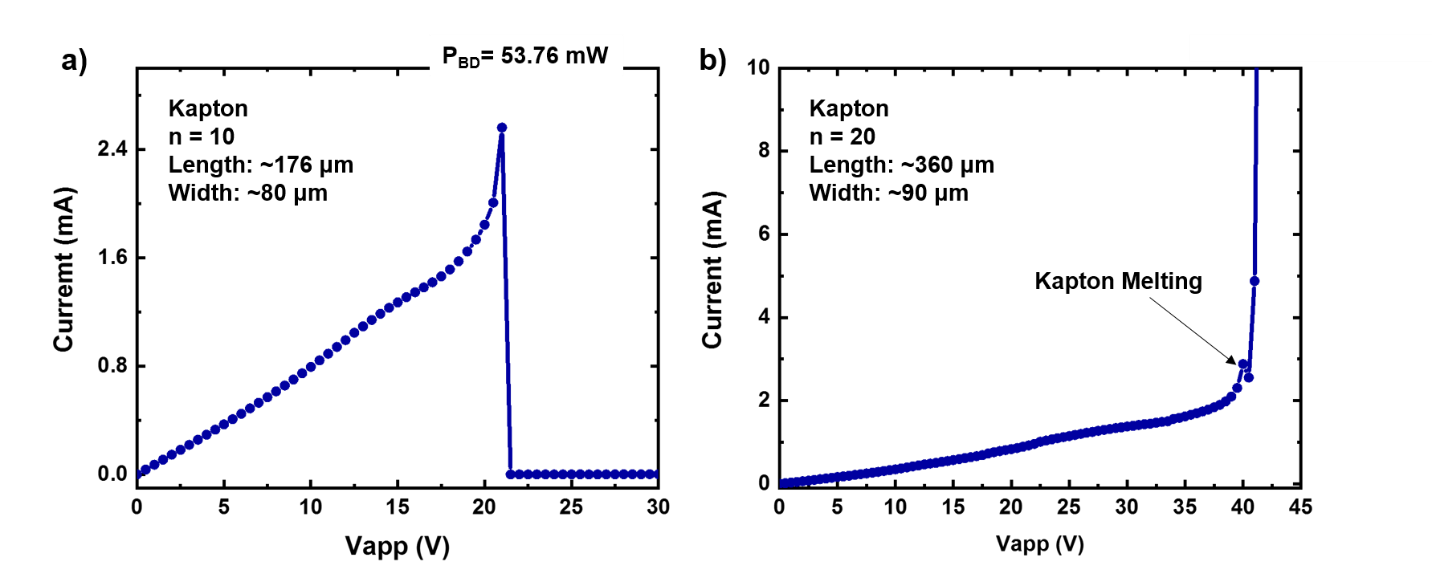

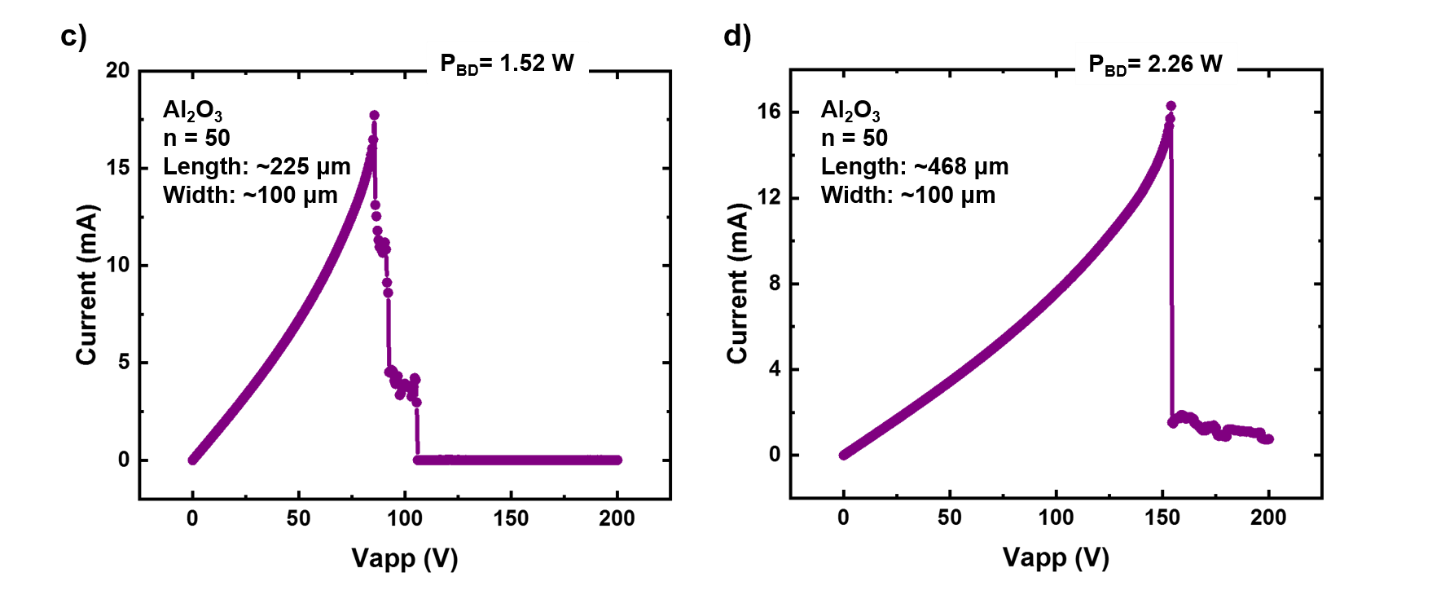

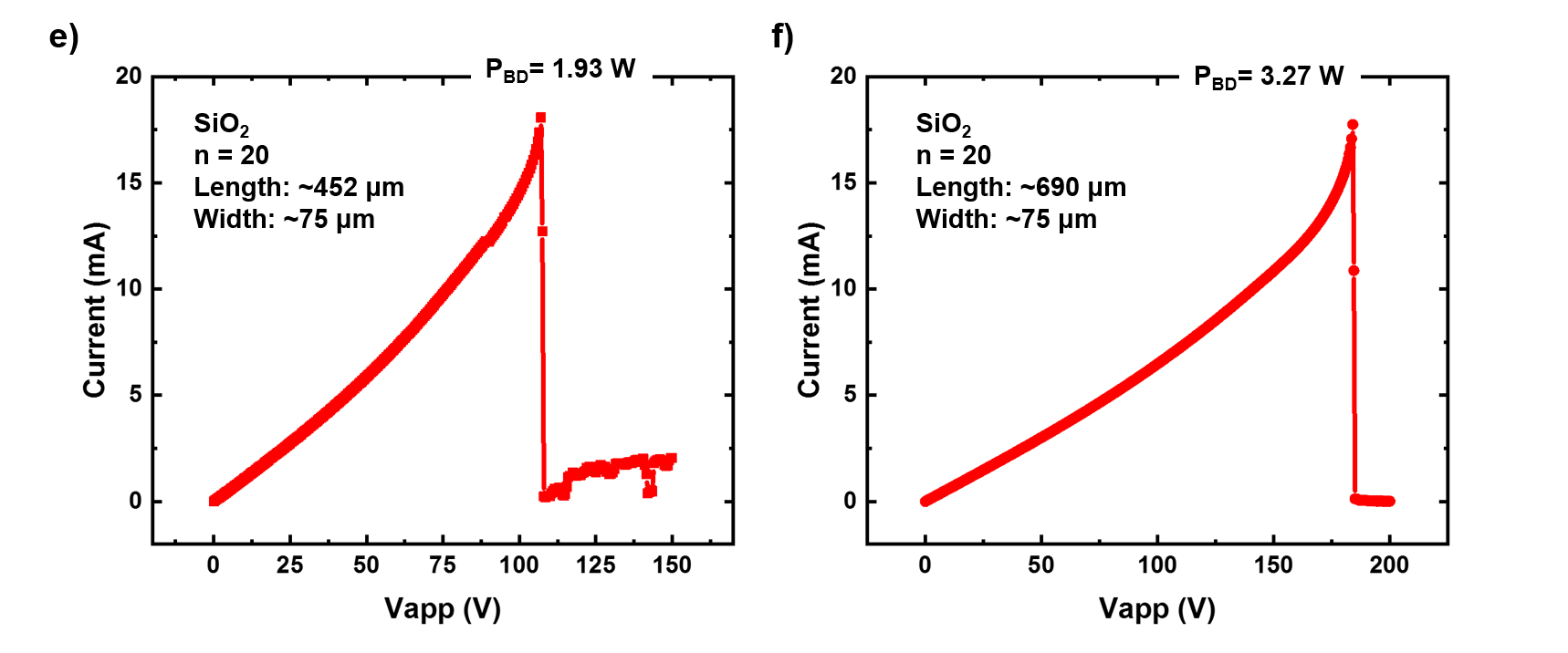
**

Figure S7: Current vs. Voltage characteristics of AJP graphene interconnects on various substrates where a-b) breakdown of Kapton^TM^, c-d) Al_2_O_3_, and e-f) breakdown of Si/SiO_2_. For b) we see that current rises till the Kapton^TM^ substrate breaks down and starts to melt at approximately 40V. The melted Kapton^TM^ results in a high conductance, and the current rises rapidly towards the instrument’s compliance limit.
